# Clinical features for diagnosis of pneumonia among adults in primary care setting: A systematic and meta-review

**DOI:** 10.1038/s41598-019-44145-y

**Published:** 2019-05-20

**Authors:** Tha Pyai Htun, Yinxiaohe Sun, Hui Lan Chua, Junxiong Pang

**Affiliations:** 1 Saw Swee Hock School of Public Health, National University of Singapore and National University Health System, Singapore, Singapore; 2 Centre for Infectious Disease Epidemiology & Research, National University of Singapore, Singapore, Singapore

**Keywords:** Respiratory signs and symptoms, Respiratory distress syndrome

## Abstract

Pneumonia results in significant morbidity and mortality worldwide. However, chest radiography may not be accessible in primary care setting. We aimed to evaluate clinical features and its diagnostic value to identify pneumonia among adults in primary care settings. Three academic databases were searched and included studies that assessed clinical predictors of pneumonia, adults without serious illness, have CXR and have conducted in primary care settings. We calculated sensitivity, specificity, positive and negative likelihood ratios, diagnostic odds ratio of each index test and the pool estimates for index tests. We identified 2,397 articles, of which 13 articles were included. In our meta-analysis, clinical features with the best pooled positive likelihood ratios were respiratory rate ≥20 min^−1^ (3.47; 1.46–7.23), temperature ≥38 °C (3.21; 2.36–4.23), pulse rate >100 min^−1^ (2.79; 1.71–4.33), and crackles (2.42; 1.19–4.69). Laboratory testing showed highest pooled positive likelihood ratios with PCT >0.25 ng/ml (7.61; 3.28–15.1) and CRP > 20 mg/l (3.76; 2.3–5.91). Cough, pyrexia, tachycardia, tachypnea, and crackles are limited as a single predictor for diagnosis of radiographic pneumonia among adults. Development of clinical decision rule that combine these clinical features together with molecular biomarkers may further increase overall accuracy for diagnosis of radiographic pneumonia among adults in primary care setting.

## Introduction

Pneumonia is an infection of the lungs caused by bacteria, virus or fungi. It is a leading cause of morbidity and mortality worldwide, especially in elder patients and patients with comorbidities. Globally, 3.2 million of the 56.4 million deaths in 2015 were cauesd by lower respiratiry tract infection^1^. The annual incidence of pneumonia was estimated at 1.07–1.2 cases per 1,000 persons per year in Europe and 16.9 cases per 1,000 persons per year in Asia^2^. Diagnosis of pneumonia in adults presenting with signs of lower respiratory tract infection is important because it requires specific treatment and follow up. Pneumonia is usually diagnosed by a combination of clinical history, physical examination and/or laboratory tests. According to most clinical guidelines globally, the supposed gold standard tool for diagnosing pneumonia is a chest X-ray (CXR) which can distinguish pneumonia from other respiratory tract infections^3,4^. Other diagnostic tests such as laboratory tests (white blood cell count (WBC), erythrocyte sedimentation rate (ESR), C-reactive protein (CRP), procalcitonin), blood culture, serology, and computed tomography scan (CT scan) have been reported with different rates of accuracy^5,6^. However, chest radiography and other diagnostic procedures, such as sputum and blood cultures, may not be accessible or not routinely measured in primary care setting for economic and logistic reasons. The superior gold standard, CT scan, is very far from available in primary care patients. Therefore, primary care physicians usually rely on patient’s medical history and physical examinations to diagnose or exclude pneumonia. Similarly, performing CXR to all suspected pneumonia cases is also challenging in the community and thereby will not always be performed for all patients. This then necessitate the need for decision aids for ordering CXR for pneumonia in the community to assess the risk more appropriately.

Several prediction rules have been identified to improve detection of pneumonia in outpatient settings^7–13^. Only one study of systematic review and meta-analysis to assess the diagnostic value of clinical features to identify pneumonia in children was conducted^14^. A systematic review and meta-analysis of the clinical features is lacking in adults. Therefore, the objective is to assess the predictive performance of clinical features associated with CXR-confirmed pneumonia compared to non-pneumonia patients in primary care settings among adults aged ≥18 years without serious illness and pre-existing immune suppression.

## Materials and Methods

### Search strategy and selection criteria

The study was performed in accordance with the recommendations of the Preferred Reporting Items for Systematic Review and Meta-Analyses (PRISMA, Appendix S1)^15^. The meta-review does not involve human participants or experiments on live vertebrates and/or higher invertebrates, only published aggregate data from the selected studies is used in the meta-analysis. The study protocol is available as Supplementary material (Appendix S2). The search strategy consists of two phases. The first phase was an extensive search using the identified index terms and keywords in three databases: PubMed, EMBASE and the Cochrane Library. The second phase was an additional search of the references of retrieved articles to find any articles that did not appear in the databases search. The keywords that were used in the search are ‘pneumonia’ or ‘community acquired pneumonia’ or ‘community-acquired pneumonia’ or ‘respiratory tract infection’ or ‘respiratory tract infections’ and ‘predictive value of tests’ or ‘sensitivity and specificity’ or ‘diagnostic test’ or ‘diagnostic tests’ or ‘medical history taking’ or ‘medical history’ or ‘physical examination’ or ‘physical examinations’ or ‘clinical laboratory techniques’ or ‘laboratory diagnoses’ or ‘laboratory examinations’ or ‘laboratory testing’ and ‘ambulatory care’ or ‘primary care’ or ‘outpatient care’ or ‘general practitioner’ or ‘emergency clinic’. The bibliographical software package, EndNote version X7 (Thomas Reuters, New York, NY, USA), was used to import references and to remove duplicates references. The remaining studies were checked against the inclusion and exclusion criteria. Two reviewers (HTP and CHL) independently screened eligibility based on title, abstracts and assessed full reports, resolving discrepancies by consensus.

Studies were selected if they were published studies that assessed clinical predictors of community-acquired pneumonia without date restrictions to maximize the search. The first search was employed on Dec 4, 2017, with an update on Mar 5, 2018. Narrative review, letters to editors, case reports and case series were excluded. Studies were included if participants aged ≥18 years without serious illness (e.g. mechanical ventilation) and pre-existing immune suppression (HIV, malnutrition, and immunosuppressant medication). To be eligible, studies had to have reference standard of CXR for diagnosing pneumonia, and have conducted in ambulatory care or primary care settings. Index tests assessed were patient’s socio-demographic, clinical signs and symptoms and laboratory tests.

### Quality assessment

After identifying studies that fulfilled the selection criteria and verifying their eligibility by reading the full articles, the quality assessment of the studies were done by using QUADAS-2^16^ as recommended by the Cochrane collaboration. Studies were assessed for selection of patient, index test, reference standard, and flow and timing. Signalling questions were made to facilitate the rating of risk of bias into low, unclear or high.

### Data extraction

The following variables were extracted from each study using pre-designed forms: study characteristics (study design, year of publication, country and setting), study population (age, number of participants recruited, prevalence of pneumonia, inclusion and exclusion criteria), reference standard (number of readers, masking and interpretation criteria), and index tests. Index tests were classified as related to socio-demographic, clinical symptoms or signs, and laboratory tests to diagnose pneumonia. Outcome data were extracted and compiled in a table by one author (HTP). After which, all extracted data were cross-checked by another author (SY) by comparing them to the original data from the selected articles.

### Data analysis

Data analysis was based on a published methodological review – a systematic review of evaluations of diagnostic and screening tests^17^. We constructed 2 × 2 tables for each study included in the review to calculate sensitivity, specificity, positive and negative likelihood ratios and diagnostic odds ratio with 95% confidence intervals (CI). The likelihood ratio indicates the value of the test for increasing certainty about a positive diagnosis. The positive likelihood ratio (LR+) is the probability of a positive result in patients with the disease, compared to the probability in patients not having the disease; while a negative likelihood ratio (LR−) is the probability of obtaining a negative test result in patients without the disease, compared to the probability in patients with the disease^18^. Clinical signs and symptoms and laboratory tests with an LR+ greater than 2.0 and an LR− less than 0.5 are clinically useful for diagnosis of pneumonia^19^. Diagnostic odds ratio is defined as the ratio of odds of the test being positive for a patient with the disease in relation to odds of the test being positive for a patient without the disease. Considering the correlation between sensitivity and specificity within and across studies, we performed bivariate model to calculate the pooled estimates of sensitivity, specificity, positive likelihood ratio, negative likelihood ratio and diagnostic odds ratio with 95% CIs. To avoid the large variances between studies, we conducted random effect meta-analysis^20^ approach as the final model. The final bivariate model was computed using the mada package in R version 3 3 4^21^. Finally, we performed the pool estimates of meta-analyses for index tests with at least four or more studies. Pool estimates for less than four studies have limited validity and hence, was excluded^22^. The index tests assessed at different thresholds were pooled together and analysed. The Summary Receiver Operating Characteristics (SROC) curve for index tests (at least four included studies) were computed using the Reitsma SROC model to obtain the summary point estimates of sensitivity and specificity as well as 95% predicting region and 95% confidence region for the summary operating point^23^.

## Results

**Figure 1:**
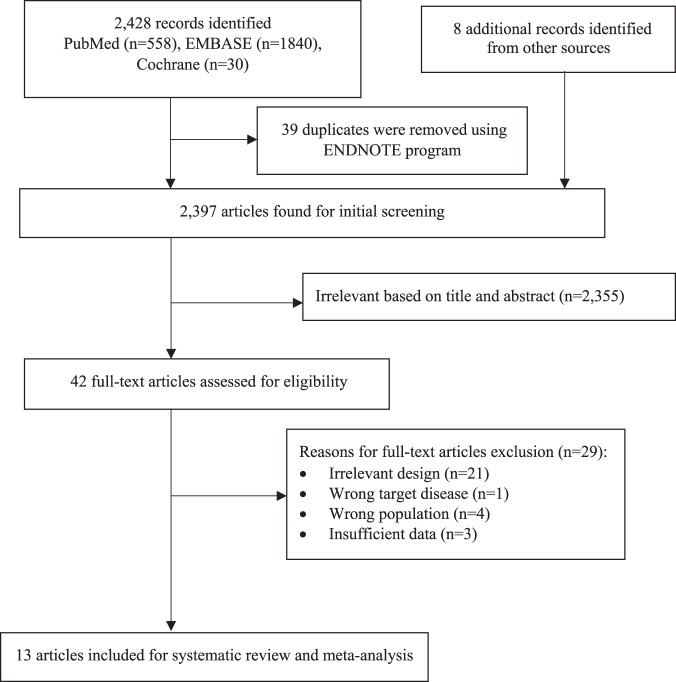
Study selection.

The selection process (PRISMA flow-diagram) is showed in Fig. 1. A total of 2,428 records were identified from the initial database search and additional records from other sources. The articles were curated using EndNote and 39 duplicates were removed. Following this, 2,397 were included for initial screening and 2,355 articles were excluded based on relevance of titles and abstracts. 42 full text articles were retrieved, reviewed and selected based on relevance and quality for eligibility. A further 29 articles were excluded because of irrelevant design (i.e. irrelevant content, unmet inclusion criteria), wrong target disease (i.e. diseases other than pneumonia e.g. influenza), wrong populations (i.e. performed in age group less than 18 years) and insufficient data. This brings the total number of included articles for this review to 13^13,24–35^.

**Figure 2:**
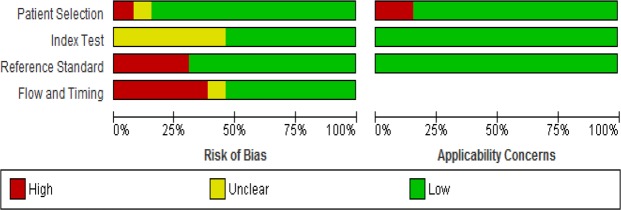
Graphical illustration of risk of bias and applicability concerns.

**Figure 3:**
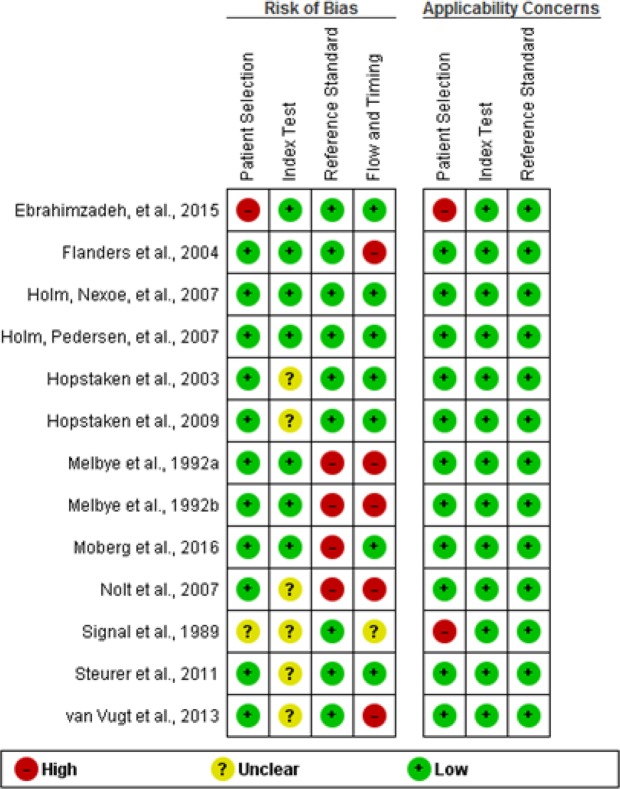
Summary of risk of bias and applicability concerns.

The methodological quality of included studies are summarised in Figs 2 and 3. In risk of bias, eleven studies had low risk in patient selection and one study had high risk for enrolling participants with confirmed diagnosis and control group without the condition. Seven studies had low risk in index test and six studies were unclear for pre-specifying of threshold of index test. Four studies had high risk in reference standard due to lack of blinding in interpretation of radiograph and extraction of data from medical records. Seven studies had low risk in flow and timing. Five studies had high risk bias due to attrition of some participants and selectively receiving the reference standards. In applicability concerns, eleven studies had low risk concerns in patient selection, whereas two studies had high risk concerns. All studies addressed low risk concerns for index test and reference standard.

The summary characteristics of the 13 included studies are shown in Table 1. A total of 11,144 participants were obtained from the studies and they are from varying locations: Iran (n = 1), USA (n = 3), Denmark (n = 2), Netherlands (n = 2), Norway (n = 2), Sweden (n = 1), Switzerland (n = 1) and Europe (n = 1). The studies were done in outpatient clinics (n = 6), emergency clinics (n = 6), primary care centres (n = 2) and GP clinic (n = 1). All the participants were adults aged ≥18 years and the sample size varied from 95 to 4,464. The study designs were prospective cohort (n = 8), case-control (n = 1), cross sectional (n = 3) and retrospective chart review (n = 1). The studies included consecutive patients with history of respiratory tract infection. In these studies, the inclusion criterion was cough alone (n = 3) and clinically suspected pneumonia (n = 10). The proportion of radiographic pneumonia in the studied populations varied from 5% to 50%.

**Table 1:** Characteristics of included studies.

Author, Year	Setting	Age, sample size	Study design	Prevalence of radiographic pneumonia	Inclusion criteria	Exclusion criteria	CXR			Index test
							Readers	Blinding	Interpretation	
Ebrahimzadeh, *et al*., 2015	Iran; Outpatient clinics and emergency clinics	≥18 years; 840	Case control study	50%	Acute respiratory symptoms with positive CXR	Acute respiratory symptoms with insignificant findings on CXR	A board certified radiologist	Yes	New consolidation without an air bronchogram, pleural effusion, abscess or empyema	Socio-demographic: Age, gender Symptoms: Cough, sputum, dyspnea, chest pain Signs: Temperature ≥38 °C, pulse rate ≥100 min^−1^ respiratory rate ≥20 min^−1^ Laboratory tests: WBC, CRP
Flanders *et al*., 2004	USA; Outpatient clinics and emergency clinics	≥18 years; 150	Prospective cohort	13.3%	Acute cough (within past 3 weeks)	Pregnancy, systematic inflammatory disorders, coexistence infections, traumas, burns, myocardial infarct or unstable angina, cancer, HIV or immunosuppressive disorders	Radiologist	Yes	Infiltrate or consolidation on chest radiograph	Socio-demographic: Age, gender, smoking Symptoms: Fever, muscle pain, fatigue, runny nose, sore throat, cough, yellow phlegm, blood in sputum, wheezing, dyspnea, chest pain Signs: Temperature ≥37.8 °C, pulse rate ≥100 min^−1^, respiratory rate ≥24 min^−1^, O_2_ saturationv ≤93%, decreased breath sounds, rales, wheezes Laboratory tests: CRP
Holm, Nexoe, *et al*., 2007	Denmark; Outpatient clinics	≥18 years; 364	Prospective cohort	13%	Clinical diagnosis of LRTI	Pregnancy, hospitalization within preceding 7 days, severe illness requiring hospitalization, former participation in the study	Experienced specialist in infectious lung disease	Yes	Transient, non-malignant infiltrate on chest film	Signs: Temperature ≥38 °C, pulse rate ≥100 min^−1^, respiratory rate ≥22 min^−1^, O_2_ saturation < 95% Laboratory tests: WBC and CRP
Holm, Pedersen, *et al*., 2007	Denmark; Outpatient clinics	≥18 years; 364	Prospective cohort	13%	Clinical diagnosis of LRTI	Pregnancy, hospitalization within preceding 7 days, severe illness requiring hospitalization, former participation in the study	Experienced specialist in infectious lung disease	Yes	Transient, non-malignant infiltrate on chest film	Laboratory tests: PCT
Hopstaken *et al*., 2003	Netherlands; Outpatient clinics	≥18 years; 243	Cross- sectional	13%	New or increasing cough, combined with other clinical characteristics	Pregnancy and lactation, allergy to penicillin, concomitant treatment with ergot alkaloids and/or terfenadine, severe clinical disease, antibiotics treatment within 14 days, hospital stay for previous 4 weeks	2 radiologists independently and 1 senior radiologist in case of disagreement	Yes	Infiltrates on chest radiograph	Socio-demographic: Age Symptoms: Dry cough, purulent sputum, dyspnea, chest pain, fever, chills, diarrhea Signs: Temperature ≥38 °C, respiratory rate > 20 min^−1^, dullness on percussion, bronchial breathing, crackles Laboratory tests: ESR, CRP
Hopstaken *et al*., 2009	Netherlands; Outpatient clinics	≥18 years; 95	Cross- sectional	11.7%	New or increasing cough, combined with other clinical characteristics	Pregnancy and lactation, allergy to penicillin, concomitant treatment with ergot alkaloids and/or terfenadine, severe clinical disease, antibiotics treatment within 14 days, hospital stay for previous 4 weeks	2 radiologists independently and 1 senior radiologist in case of disagreement	Yes	Infiltrates on chest radiograph	Signs: Temperature ≥38 °C Laboratory tests: CRP, LBP, fibrinogen
Melbye *et al*., 1992	Norway; Municipal emergency clinic	≥18 years; 402	Prospective cohort	41% (21 out of 51 CXR patients)	Symptoms of respiratory tract or throat infection	Pregnancy, severe dyspnea patients	2 radiologists and 1 senior chest physician independently	NR	A density on chest film	Typical symptoms: Dry cough, purulent sputum, dyspnea, chest pain, fever, chills Atypical symptoms: Fatigue, myalgia/arthralgia, coryza, sore throat Signs: Wheezes, crackles, decreased breath sounds, dullness to percussion
Melbye *et al*., 1992	Norway; Municipal emergency clinic	≥18 years; 402	Prospective cohort	41% (21 out of 51 CXR patients)	Symptoms of respiratory tract or throat infection	Pregnancy, severe dyspnea patients	2 radiologists and 1 senior chest physician independently	NR	A density on chest film	Laboratory tests: ESR, WBC and CRP
Moberg *et al*., 2016	Sweden; Primary care centres	≥18 years; 103	Prospective cohort	45%	Respiratory tract infection symptoms for 24 hour	Pregnancy, COPD, received antibiotics less than 2 weeks, patients living in nursing home	Radiologists on duty and a board certified radiologist	No	Infiltrates on chest radiograph	Socio-demographic: Gender, smoking Symptoms: Chest pain Signs: Temperature > 38 °C, pulse rate > 100 min^−1^, respiratory rate > 20 min^−1^, O_2_ saturation < 95% crackles, rales, decreased breath sounds, dullness on percussion Laboratory tests: WBC, CRP
Nolt *et al*., 2007	USA; Emergency clinics	≥18 years; 4464	Retrospective charts review	12%	Acute cough illness	Any visits without a chief complaint of cough	Radiography notes were abstracted by research coordinators	NR	Haziness, density, consolidation, inflammation, infiltration or acute pulmonary abnormality in radiology report	Socio-demographic: Age, smoking Signs: Temperature ≥100.4 °F, pulse rate >100 min^−1^, respiratory rate ≥20 min^−1^, O_2_ saturation <95%
Signal *et al*., 1989	USA; Emergency clinics	≥18 years; 255	Prospective cohort	15.6%	Patients who perform chest radiography	Critically ill patients	A board certified radiologist and final typed report was reviewed by the investigators	NR	Infiltrates on chest radiograph	Socio-demographic: Age, gender Symptom: Cough, chest pain and dyspnea Signs: Crackles, wheezes, tachycardia, tachypnea
Steurer *et al*., 2011	Switzerland; GP clinics	≥18 years; 642	Prospective cohort	20.5%	New or worsening cough for 24 hour, with increased body temperature	Pregnancy, chronic lung diseases, HIV patients taking oral steroid, on chemotherapy, organ transplantation, mental disorder	Radiologists	Yes	Shadow on radiograph	Socio-demographic: Age, gender, smoking Symptoms: Cough, fever, dyspnea, wheezing, chest pain, muco-purulent sputum, bloody sputum Signs: Decreased breath sound, bronchial breath sound, dullness on percussion Laboratory tests: CRP
van Vugt *et al*., 2013	Europe; Primary care centres	≥18 years; 2820	Cross sectional	5%	Acute cough	No chest radiograph performed or insufficient quality of radiograph	Radiologists	Yes	Diagnosis by selecting one of the following fixed option responses such as normal chest radiograph, acute bronchitis, pneumonia, or other diagnosis	Socio-demographic: Age, gender, smoking Symptoms: Cough, phlegm, dyspnea, runny nose, fever, chest pain, diarrhea Signs: Diminished vesicular breath sound, crackles, temperature > 37.8 °C, pulse rate > 100 min^−1^, respiratory rate > 24 min^−1^ Laboratory tests: PCT and CRP

COPD = chronic obstructive pulmonary disease. CRP = C-reactive protein. CXR = chest X-ray. ESR = erythrocyte sedimentation rate. HIV = human immunodeficiency virus. LBP = lipopolysaccharide binding protein.

LRTI = lower respiratory tract infection. NR = not reported. PCT = procalcitonin. WBC = white blood cell count.

A total of 25 different clinical history and features studied for their accuracy in diagnosis of radiographic pneumonia: related to socio-demographic (n = 3), symptoms (n = 13), signs (n = 9). The 13 included papers comprised 40 clinical index tests. Of the 40 index tests, the most frequently assessed index tests were: history of fever (n = 5), cough (n = 7), sputum (n = 6), dyspnea (n = 7), chest pain (n = 8), crackles (n = 7), elevated temperature (n = 9), increased pulse rate (n = 6), respiratory rate (n = 7), and decreased breath sounds (n = 5). Different thresholds were used to measure age, temperature, pulse rate, respiratory rate, and O2 saturation. Six different laboratory tests (white blood cell count (WBC), erythrocyte sedimentation rate (ESR), C-reactive protein (CRP), procalcitonin (PCT), lipopolysaccharide binding protein (LBP) and fibrinogen were used to examine the diagnostic value of radiographic pneumonia. CRP was the most frequently assessed index test (n = 10), however, each with a different standard.

**Figure 4:**
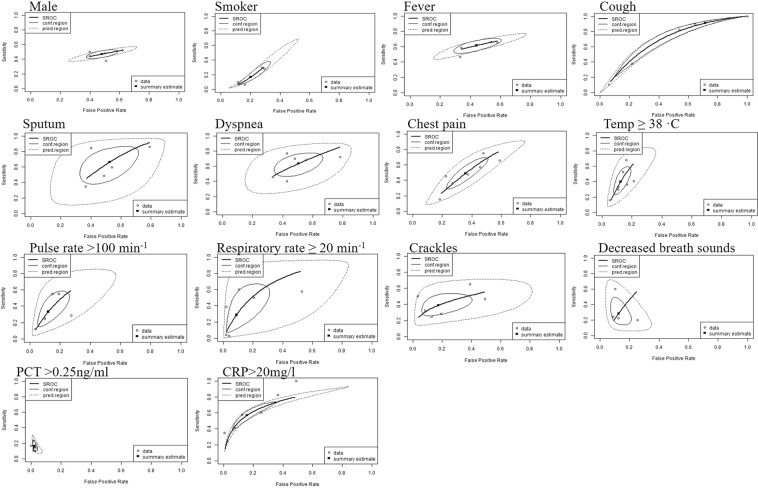
Summary ROC plot for socio-demographic, symptoms, signs and laboratory tests.

The diagnostic performance measures (sensitivity, specificity, positive and negative likelihood ratios, and diagnostic odds ratio) of each index test was prescribed detail in Supplementary material (Appendix S3). Pooled estimates for gender (male), smoker, fever (≥37.5 °C), cough, sputum, dyspnoea, chest pain, temperature, pulse rate, respiratory rate, crackles, decreased breath sounds, PCT, and CRP were obtained by meta-analysis. The summary estimates of each index test’s diagnostic performance measures (sensitivity, specificity, positive, negative likelihood ratio and diagnostic odds ratio) are shown in Table 2. Among the index tests, cough had high sensitivity 0.91 (0.36–0.99) but had low specificity 0.28 (0.03–0.83). We would estimate that 91% of patients with radiographic pneumonia would have symptoms of cough. Some index tests had specificities higher than 0.80, such as temperature ≥ 38 °C 0.88 (0.82–0.91), pulse rate >100 min^−1^ 0.88 (0.77–0.94), respiratory rate ≥20 min^−1^ 0.91 (0.75–0.97), crackles 0.83 (0.65–0.92), decreased breath sounds 0.87 (0.81–0.92), PCT >0.25 ng/ml 0.98 (0.96–0.99) and CRP >20 mg/l 0.84 (0.7–0.93). The clinical features with pooled estimates of significantly high positive likelihood ratios as defined by LR+ >2.0 were temperature ≥ 38 °C, pulse rate >100 min^−1^, respiratory rate ≥20 min^−1^, crackles, PCT >0.25 ng/ml and CRP > 20 mg/l. The clinical features with pooled estimates of significantly high negative likelihood ratio (LR− <0.5) was cough. The highest positive likelihood ratio observed was PCT (7.61) followed by CRP (3.76), respiratory rate ≥20 min^−1^ (3.47), temp ≥ 38 °C (3.21), pulse rate >100 min^−1^ (2.79), and crackles (2.42). Overall, based on diagnostic odds ratio, cough, crackles, respiratory rate ≥20 min^−1^, fever with temperature ≥ 38 °C, pulse rate >100 min^−1^, decreased breath sounds, CRP and PCT were potential useful diagnostic indicators of pneumonia. The SROC plot of summary point estimates of sensitivity and specificity with 95% confidence region and 95% prediction region are shown in Fig. 4.

**Table 2:** Summary estimates of diagnostic performance measures of each index test assesses in four studies or more.

Factor	Number of studies	Total population	Sensitivity (95% CI)	Specificity (95% CI)	Positive likelihood ratio (95% CI)	Negative likelihood ratio (95% CI)	Diagnostic odds ratio (95% CI)
**Socio-demographic**							
Male	5	4,549	0.47 (0.42–0.52)	0.52 (0.43–0.6)	0.98 (0.86–1.14)	1.03 (0.91–1.18)	0.96 (0.73–1.25)
Smoker	4	3,707	0.17 (0.08–0.33)	0.80 (0.70–0.87)	0.84 (0.56–1.15)	1.03 (0.94, 1.09)	0.82 (0.52–1.22)
**Symptoms**							
Fever	4	3,849	0.61 (0.53–0.69)	0.56 (0.43–0.68)	1.41 (1.15–1.78)	0.70 (0.59–0.82)	2.06 (1.4–2.91)
Cough^*^	6	4,945	0.91 (0.36–0.99)	0.28 (0.03–0.83)	1.36 (1.03–2.10)	0.36 (0.15–0.78)	4.23 (2.44–6.83)
Sputum^**^	5	4,690	0.66 (0.44–0.83)	0.48 (0.32–0.64)	1.27 (0.90–1.72)	0.72 (0.39–1.13)	1.95 (0.79–4.04)
Dyspnea	6	4,946	0.63 (0.50–0.75)	0.49 (0.36–0.63)	1.27 (0.99–1.63)	0.75 (0.53–1.01)	1.77 (0.98–2.97)
Chest pain	7	5,044	0.49 (0.32–0.66)	0.64 (0.52–0.75)	1.37 (1.14–1.60)	0.79 (0.62–0.93)	1.76 (1.23–2.44)
**Signs**							
Temp ≥ 38 °C^†^	7	4,593	0.40 (0.26–0.56)	0.88 (0.82–0.91)	3.21 (2.36–4.23)	0.68 (0.53–0.82)	4.80 (2.96–7.38)
Pulse rate > 100 min^−1‡^	5	4,256	0.33 (0.18–0.53)	0.88 (0.77–0.94)	2.79 (1.71–4.33)	0.76 (0.57–0.90)	3.78 (1.99–6.57)
Respiratory rate ≥ 20 min^−1¥^	6	4,468	0.29 (0.10–0.59)	0.91 (0.75–0.97)	3.47 (1.46–7.23)	0.77 (0.50–0.95)	4.74 (1.6–11.00)
Crackles^¶^	6	3,671	0.39 (0.28–0.51)	0.83 (0.65–0.92)	2.42 (1.19–4.69)	0.75 (0.61–0.91)	3.34 (1.13–7.06)
Decreased breath sounds	4	3,394	0.28 (0.16–0.45)	0.87 (0.81–0.92)	2.43 (0.98–4.87)	0.82 (0.61–1.00)	3.17 (0.97–7.78)
**Lab investigations**							
PCT > 0.25 ng/ml^$^	4	6,042	0.16 (0.11–0.22)	0.98 (0.96–0.99)	7.61 (3.28–15.1)	0.86 (0.79–0.92)	8.98 (3.59–18.8)
CRP > 20 mg/l^§^	9	9,476	0.57 (0.42–0.70)	0.84 (0.70–0.93)	3.76 (2.30–5.91)	0.52 (0.42–0.63)	7.21 (5.08–9.94)

*Dry cough in one study is included. **Yellowish purulent sputum in three studies are included. ^†^Temperature ≥37.8 °C in two studies are included. ^‡^Pulse rate ≥100 min^−1^ in one study is included. ^¥^Respiratory rate ≥22 min^−1^ in one study, respiratory rate ≥24 min^−1^ in two studies are included. ^¶^Rales in two studies are included. ^$^PCT >0.50 ng/ml in two studies are included. ^§^CRP >50 mg/l in two studies and CRP > 100 mg/l in three studies are included.

## Discussion

Clinicians have traditionally used certain clinical signs and symptoms to diagnose pneumonia in the community. We aimed to assess the clinical predictors for diagnosis of pneumonia in adults to complement the clinical judgement for the need of CXR in a primary care setting, where CXR may not be readily available. The results of the pooled diagnostic odds ratio for clinical signs and laboratory tests were promising in our findings. However, the pooled diagnostic odds ratio for socio-demographic and symptoms were not ideal as predictors except for cough.

Our meta-analysis showed that individual clinical history and symptoms do not have adequate discriminatory power except cough to diagnose pneumonia among adults in primary care setting. This is consistent with previous study showing that no clinical symptoms is sufficient on its own for diagnosis of radiographic pneumonia among children under five years old^14^. Consistent with the previous analyses, cough was a poorly specific indicator of pneumonia, assuming that patients visiting to clinic with symptoms of cough would unlikely to have pneumonia^14,36^. However, there is likely an overestimation of cough because it was part of the inclusion criteria for most of the studies. Thus, likely resulting in cough having a good pooled negative likelihood ratio and high diagnostic odds ratio in our study, Respiratory rate (one of the criteria to classify pneumonia) was one of the two most useful predictors among the clinical signs, beside temperature ≥38 °C based on diagnostic odds ratio. Fast breathing had highest specificity, therefore it might be useful clinically to identify patients without fast breathing would be unlikely to have pneumonia. There was evidence that an adult with a respiratory rate of over 20 per minute is probably unwell and an adult with a respiratory rate of over 24 breaths per minute is likely to be critically ill^37^. Pyrexia was the next most useful predictor, and followed by tachycardia. These findings are similar to the clinical decision rule of a published study^9^, that ordered CXR only for patients with at least one abnormal vital signs (i.e. temperature greater than 37.8 °C, respiratory rate greater than 20 breaths per minute, or pulse rate greater than 100 beats per minute). Consistent with other studies^7,11^, auscultation sounds such as crackles was shown as predictor of pneumonia in our study. Moreover, the predictors in our findings were also found in Heckerling clinical decision rules for pulmonary infiltrates. The rule identified five key predictors for pneumonia: temperature greater than 37.8 °C, pulse rate greater than 100 beats per minute, crackles, decreased breath sounds, and absence of asthma^11^. In addition, fever, tachycardia and crackles were observed to be useful as part of the predictions models externally validated for pneumonia in primary care^38^.

In our results, biomarker such as PCT and CRP were the strongest predictors among all variables tested and had significant discriminating power than clinical signs and symptoms for pneumonia. PCT, a marker of sepsis, strongly correlated with bacteria load^39^ and the severity of infection^40^. In addition, elevated PCT levels point towards bacterial infection rather than viral infection^41^. There is some evidence that PCT >0.25 ng/ml reflects a typical bacterial aetiology^42^. This evidence is in line with our result demonstrating that PCT > 0.25 ng/ml was able to predict pneumonia. On the other hand, PCT has been most frequently studied with regard to its prognostic value and correlation with disease severity^42,43^^,^ in patients with pneumonia. Notably, PCT has been regarded as a prognostic rather than diagnostic factor in adult patients with community-acquired pneumonia^42^. CRP is a widely used point of care test in ambulatory care. CRP has been studied as a screening device for inflammation, and as a marker of bacterial infection^44,45^. Our result revealed that the diagnostic role of CRP > 20 mg/l has value in ruling in pneumonia. This finding is similar to previous systematic reviews showing that pneumonia is ruled out if CRP below 20 mg/l^44,46^. Moreover, it also seems that CRP test cannot be used as a stand-alone diagnostic test for pneumonia. Current evidences have shown that adding CRP value to basic signs and symptoms models in diagnosing pneumonia improved diagnostic discrimination of adult patients in primary care^10,47^.

There are a number of limitations in this review. Firstly, there was heterogeneity among the selected studies in terms of inclusion criteria, chest radiograph (interpretation criteria and lack of blinding), inconsistencies in the reporting of clinical features across different studies and the prevalence of pneumonia. Sensitivity and specificity values are highly dependent on the prevalence of the pnemonia in the respective population of different studies. Moreover, the time between potential exposure to infection and the point when the test gives an accurate result was not clearly reported in the studies. Bivariate random effects model was used to account for heterogeneity between the studies. Secondly, a small number of variables did not allow for meta-analysis to be conducted to investigate the tests’ accuracies. Thirdly, it is possible that there are some relevant studies which were not published, resulting in potential publication bias. In addition, only studies published in English were included in our review which may have resulted in limited generalizability. Finally, our findings may also have limited applicability in low- or middle-income countries, since all the selected studies except one study^24^ were conducted in high income countries. Moreover, the study only focused on the predictive nature of the variables singly and potentially, performance of the variables may be improved but likely to a limited extent with more than one clinical signs and symptoms as covariables in the model.

The findings of this review suggest that individual clinical symptom (cough) and clinical signs (pyrexia, tachycardia, tachypnea, and crackles) are associated to pneumonia but limited as a single predictor for diagnosis of radiographic pneumonia. The combination of these clinical features in decision rule might indeed enhance the overall diagnostic performance of individual symptoms and signs. Future high quality and large-scale case-control studies using the clinical data relevant to the population of interest is necessary to assess the combination with the clinical features identified in this review, and to propose a practical scoring system to aid clinical judgement for ordering of CXR to confirm pneumonia. Moreover, the combination of these clinical features together with molecular biomarkers is likely to further add value to the overall diagnostic accuracy.

## Supplementary information

Supplementary Information
