# Supplementary material for: Clinical features for diagnosis of pneumonia among adults in primary care setting: A systematic and meta-review
Source: Sci Rep. 2019 May 20;9:7600. doi: 10.1038/s41598-019-44145-y (PMC6527561; doi:10.1038/s41598-019-44145-y)
Supplement: Supplementary file 1 — Supplementary Information [file 41598_2019_44145_MOESM1_ESM.pdf]

Clinical features for diagnosis of pneumonia among adults in primary care setting: A systematic and meta-review

Tha Pyai HTUN, MBBS, MPH.

Research Associate

Saw Swee Hock School of Public Health

National University of Singapore and National University Health System, Singapore

Yinxiaohe SUN, MSc

Research Assistant

Saw Swee Hock School of Public Health

National University of Singapore and National University Health System, Singapore

Hui Lan CHUA, BSc

Research Assistant

Saw Swee Hock School of Public Health

National University of Singapore and National University Health System, Singapore

Junxiong PANG, MSc, PhD

Assistant Professor

Saw Swee Hock School of Public Health

National University of Singapore and National University Health System, Singapore

Keywords: Clinical features, Pneumonia, Diagnosis, Primary Care, Adults

Short title: Clinical features and adult pneumonia in primary care setting

Main Correspondence:

PANG Junxiong, PhD

Assistant Professor, Centre for Infectious Disease Epidemiology & Research,

Saw Swee Hock School of Public Health and National University Health System,

Tahir Foundation Building, Level 9, 12 Science Drive 2, Singapore 117549;

E-mail: ephpjv@nus.edu.sg; pangv@hotmail.com

Tel: +6566014993

PRISMA 2009 Checklist

Section/topic	#	Checklist item	Reported on page #
**TITLE**			
Title	1	Identify the report as a systematic review, meta-analysis, or both.	1
**ABSTRACT**			
Structured summary	2	Provide a structured summary including, as applicable: background; objectives; data sources; study eligibility criteria, participants, and interventions; study appraisal and synthesis methods; results; limitations; conclusions and implications of key findings; systematic review registration number.	3 & 4
**INTRODUCTION**			
Rationale	3	Describe the rationale for the review in the context of what is already known.	5
Objectives	4	Provide an explicit statement of questions being addressed with reference to participants, interventions, comparisons, outcomes, and study design (PICOS).	5
**METHODS**			
Protocol and registration	5	Indicate if a review protocol exists, if and where it can be accessed (e.g., Web address), and, if available, provide registration information including registration number.	6
Eligibility criteria	6	Specify study characteristics (e.g., PICOS, length of follow-up) and report characteristics (e.g., years considered, language, publication status) used as criteria for eligibility, giving rationale.	6,7
Information sources	7	Describe all information sources (e.g., databases with dates of coverage, contact with study authors to identify additional studies) in the search and date last searched.	6
Search	8	Present full electronic search strategy for at least one database, including any limits used, such that it could be repeated.	6
Study selection	9	State the process for selecting studies (i.e., screening, eligibility, included in systematic review, and, if applicable, included in the meta-analysis).	6,7
Data collection process	10	Describe method of data extraction from reports (e.g., piloted forms, independently, in duplicate) and any processes for obtaining and confirming data from investigators.	6,7
Data items	11	List and define all variables for which data were sought (e.g., PICOS, funding sources) and any assumptions and simplifications made.	6,7
Risk of bias in individual studies	12	Describe methods used for assessing risk of bias of individual studies (including specification of whether this was done at the study or outcome level), and how this information is to be used in any data synthesis.	7
Summary measures	13	State the principal summary measures (e.g., risk ratio, difference in means).	8
Synthesis of results	14	Describe the methods of handling data and combining results of studies, if done, including measures of consistency (e.g., I^2) for each meta-analysis.	6,7,8

PRISMA 2009 Checklist

Section/topic	#	Checklist item	Reported on page #
Risk of bias across studies	15	Specify any assessment of risk of bias that may affect the cumulative evidence (e.g., publication bias, selective reporting within studies).	7
Additional analyses	16	Describe methods of additional analyses (e.g., sensitivity or subgroup analyses, meta-regression), if done, indicating which were pre-specified.	8
**RESULTS**			
Study selection	17	Give numbers of studies screened, assessed for eligibility, and included in the review, with reasons for exclusions at each stage, ideally with a flow diagram.	8,9
Study characteristics	18	For each study, present characteristics for which data were extracted (e.g., study size, PICOS, follow-up period) and provide the citations.	12-18, S3
Risk of bias within studies	19	Present data on risk of bias of each study and, if available, any outcome level assessment (see item 12).	10,11,12
Results of individual studies	20	For all outcomes considered (benefits or harms), present, for each study: (a) simple summary data for each intervention group (b) effect estimates and confidence intervals, ideally with a forest plot.	12-18, S3
Synthesis of results	21	Present results of each meta-analysis done, including confidence intervals and measures of consistency.	19, 20
Risk of bias across studies	22	Present results of any assessment of risk of bias across studies (see Item 15).	10,11,12
Additional analysis	23	Give results of additional analyses, if done (e.g., sensitivity or subgroup analyses, meta-regression [see Item 16]).	20-23
**DISCUSSION**			
Summary of evidence	24	Summarize the main findings including the strength of evidence for each main outcome; consider their relevance to key groups (e.g., healthcare providers, users, and policy makers).	24,25
Limitations	25	Discuss limitations at study and outcome level (e.g., risk of bias), and at review-level (e.g., incomplete retrieval of identified research, reporting bias).	25, 26
Conclusions	26	Provide a general interpretation of the results in the context of other evidence, and implications for future research.	26
**FUNDING**			
Funding	27	Describe sources of funding for the systematic review and other support (e.g., supply of data); role of funders for the systematic review.	27, 28

From: Moher D, Liberati A, Tetzlaff J, Altman DG, The PRISMA Group (2009). Preferred Reporting Items for Systematic Reviews and Meta-Analyses: The PRISMA Statement. PLoS Med 6(7): e1000097. doi:10.1371/journal.pmed1000097

For more information, visit: www.prisma-statement.org.

Clinical features for diagnosis of pneumonia among adults in primary care setting: A systematic and meta-review

Study Protocol

OBJECTIVES

The purpose of this study is to assess the predictive performance of clinical features associated with chest X-ray radiologically (CXR)-confirmed pneumonia compared to non-pneumonia patients in primary care settings among adults aged ⩾ 18 years without serious illness and pre-existing immune suppression.

Primary objectives

1. To determine the strength of association between specific clinical signs and symptoms and the occurrence of CXR-confirmed pneumonia in primary care settings.
2. To determine the predictive performance of these specific clinical signs and symptoms to predict CXR-confirmed pneumonia in primary care settings.

METHODS

Study selection

Inclusion criteria:

- Study design: Studies will be selected if they are published studies that assessed clinical predictors of community-acquired pneumonia without date restrictions and have conducted in ambulatory care or primary care settings.
- Population of interest: Participants aged ⩾ 18 years without serious illness (e.g. mechanical ventilation) and pre-existing immune suppression (HIV, malnutrition, and immunosuppressant medication)
- Interventions: studies with clinical signs and symptoms associated with the reference standard of CXR for diagnosing pneumonia.
- Comparator: studies with control group which included patients without pneumonia confirmed by CXR.
- Outcomes: studies with CXR-confirmed pneumonia as outcome

Exclusion criteria:

- Studies lacking components mentioned above
- Narrative review, letters to editors, case reports and case series will be excluded

Data abstraction

The following information will be collected where available:

- Name of trial/author/journal
- Year of publication
- Study setting (country, clinical setting)
- Study design
- Randomization process, if any
- Diagnostic test for pneumonia
- Sample size
- Characteristics of study participants
- Control group
- Study results (clinical signs and symptoms) based on the outcomes of interest

Search strategy

The search strategy consists of two phases. The first phase will be an extensive search using the identified index terms and keywords in three databases: PubMed, EMBASE and the Cochrane Library. The second phase will be an additional search of the references of retrieved articles to find any articles that did not appear in the databases search. Search will be limited to English language articles. The bibliographical software package, EndNote version X7 (Thomas Reuters, New York, NY, USA), will be used to import references and to remove duplicates references. The remaining studies will be checked against the inclusion and exclusion criteria. Two reviewers will independently screen eligibility based on title, abstracts and assessed full reports, resolving discrepancies by consensus.

Analysis plan

- 2×2 tables for each study will be constructed to calculate sensitivity, specificity, positive and negative likelihood ratios and diagnostic odds ratio with 95% confidence intervals (CI).
- When four or more studies of specific clinical signs and symptoms are available that report on the same outcome of interest, the results will be pooled.
- Random effects meta-analysis will be used for all estimates of effect
- Considering the correlation between sensitivity and specificity within and across studies, bivariate model will be performed to calculate the pooled estimates of sensitivity, specificity, positive likelihood ratio, negative likelihood ratio and diagnostic odds ratio with 95% CIs.
- The final bivariate model will be computed using the mada package in R version 3·3·4.
- Pool estimates for less than four studies have limited validity and hence, was excluded.
- The index tests assessed at different thresholds will be pooled together and analysed.
- The Summary Receiver Operating Characteristics (SROC) curve for index tests (at least four included studies) will be computed using the Reitsma SROC model to obtain the summary point estimates of sensitivity and specificity as well as 95% predicting region and 95% confidence region for the summary operating point.
- Heterogeneity will be assessed using Chi-squared test available in RevMan with $p<0.05$ used to determine statistical significance.
- If >10 studies are available for an adherence intervention, funnel plots will be used to assess publication bias.
- The quality assessment of the studies will be done by using QUADAS-2 as recommended by the Cochrane collaboration. Studies will be assessed for selection of patient, index test, reference standard, and flow and timing. Signalling questions will be made to facilitate the rating of risk of bias into low, unclear or high.

Data management

Outcome data will be extracted and compiled in a table by one author. After which, all extracted data will be cross-checked independently by another author by comparing them to the original data from the selected articles. However objections to the data will be addressed and resolved between all co-authors by consensus before proceeding with the analysis. The initial data analysis will address only the objectives specified above. Preliminary findings from these analyses will be circulated among contributing investigators for their comments and suggestions about further analysis.

S3 Appendix. Diagnostic performance measures of each index test assesses in included studies

Socio-demographic

Author, Year	Index test	Total population	True positive	False negative	False positive	True negative	Sensitivity (95% CI)	Specificity (95% CI)	Positive likelihood ratio (95% CI)	Negative likelihood ratio (95% CI)	Diagnostic odds ratio (95% CI)
**Age**											
Hopstaken et al., 2003	Age ≥ 65	243	11	21	59	152	0·34 (0·20-0·52)	0·72 (0·66-0·78)	1·23 (0·72-2·08)	0·91 (0·70-1·19)	1·3
**Gender**											
Ebrahimzadeh, et al., 2015	Male	840	220	200	227	193	0·52 (0·48-0·57)	0·46 (0·41-0·51)	0·97 (0·85-1·1)	1·04 (0·90-1·20)	0·9
Flanders et al., 2004	Male	168	10	10	59	89	0·50 (0·30-0·70)	0·60 (0·52-0·68)	1·25 (0·78-2·03)	0·83 (0·53-1·31)	1·5
Moberg et al., 2016	Male	100	17	28	28	27	0·38 (0·25-0·52)	0·49 (0·36-0·62)	0·74 (0·47-1·17)	1·27 (0·89-1·80)	0·6
Steurer et al., 2011	Male	621	61	66	247	247	0·48 (0·39-0·57)	0·50 (0·46-0·54)	0·96 (0·79-1·18)	1·04 (0·86-1·26)	0·9
van Vugt et al., 2013	Male	2820	62	78	1066	1614	0·44 (0·36-0·53)	0·60 (0·58-0·62)	1·11 (0·92-1·35)	0·93 (0·80-1·08)	1·2
**Smoking**											
Flanders et al., 2004	Smoker	168	2	18	17	131	0·1 (0·03-03)	0·89 (0·82-0·93)	0·87 (0·23-3·49)	1·02 (0·87-1·19)	0·9
Moberg et al., 2016	Smoker	100	3	42	9	46	0·07 (0·02-0·18)	0·84 (0·72-0·91)	0·41 (0·12-1·42)	1·12 (0·97-1·28)	0·4
Steurer et al., 2011	Smoker	621	36	91	145	349	0·28 (0·21-0·37)	0·71 (0·66-0·75)	0·97 (0·71-1·32)	1·01 (0·90-1·15)	0·9
van Vugt et al., 2013	Smoker	2818	42	98	737	1941	0·30 (0·23-0·38)	0·72 (0·71-0·74)	1·09 (0·84-1·42)	0·97 (0·86-1·08)	1·1

Symptoms

Author, Year	Index test	Total population	True positive	False negative	False positive	True negative	Sensitivity (95% CI)	Specificity (95% CI)	Positive likelihood ratio (95% CI)	Negative likelihood ratio (95% CI)	OR
**Fever**											
Flanders et al., 2004	Fever	168	13	7	86	62	0·65 (0·43-0·82)	0·42 (0·34-0·50)	1·12 (0·79-1·59)	0·84 (0·45-0·51)	1·3
Hopstaken et al., 2003	Fever	243	15	17	70	141	0·47 (0·31-0·64)	0·67 (0·60-0·73)	1·41 (0·93-2·14)	0·80 (0·57-1·12)	1·8
Melbye et al., 1992	Fever	420	NA	NA	NA	NA	0·65	0·51	1·30 (0·9-2·0)	NA	NA
Steurer et al., 2011	Fever	621	84	43	266	228	0·66 (0·58-0·74)	0·46 (0·42-0·51)	1·23 (1·06-1·42)	0·73 (0·95-1·11)	1·7
van Vugt et al., 2013	Fever	2817	82	58	907	1770	0·59 (0·50-0·66)	0·66 (0·64-0·68)	1·73 (1·49-2·01)	0·63 (0·51-0·76)	2·8
**Chills**											
Hopstaken et al., 2003	Chills	243	22	10	100	111	0·69 (0·51-0·82)	0·53 (0·46-0·59)	1·45 (1·10-1·91)	0·59 (0·35-1·00)	2·4
Melbye et al., 1992	Chills	420	NA	NA	NA	NA	0·80	0·67	1·9 (1·2-3·1)	NA	NA
**Cough**											
Ebrahimzadeh, et al., 2015	Cough	840	376	44	272	148	0·89 (0·86-0·92)	0·35 (0·31-0·40)	1·38 (1·28-1·49)	0·30 (0·22-0·41)	4·6
Flanders et al., 2004	Cough	168	20	0	148	0	1·0 (0·84-1·0)	0·00	1·00	NA	NA
Hopstaken et al., 2003	Cough (dry)	243	12	20	46	165	0·38 (0·23-0·55)	0·78 (0·72-0·83)	1·72 (1·03-2·88)	0·80 (0·61-1·06)	2·2
Melbye et al., 1992	Cough (dry)	420	NA	NA	NA	NA	0·40	0·81	2·2 (1·1-4·1)	NA	NA
Signal et al., 1989	Cough	255	33	7	116	99	0·83 (0·68-0·91)	0·46 (0·40-0·53)	1·53 (1·27-1·85)	0·38 (0·19-0·76)	4·02
Steurer et al., 2011	Cough	621	13	114	30	464	0·10 (0·06-0·17)	0·94 (0·91-0·96)	1·69 (0·91-3·14)	0·96 (1·02-0·89)	1·8
van Vugt et al., 2013	Cough	2818	140	0	2678	0	1·00 (0·97-1·0)	0·00	1·00	NA	NA
**Sputum**											
Ebrahimzadeh, et al., 2015	Sputum	840	354	66	168	252	0·84 (0·81-0·87)	0·60 (0·55-0·65)	2·11 (1·86-2·39)	0·26 (0·21-0·33)	8·0
Flanders et al., 2004	Yellow phlegm	168	7	13	54	94	0·35 (0·18-0·57)	0·64 (0·56-0·71)	0·96 (0·51-1·81)	1·02 (0·73-1·44)	0·9
Hopstaken et al., 2003	Sputum (purulent)	243	19	13	114	97	0·59 (0·42-0·74)	0·46 (0·39-0·53)	1·10 (0·80-1·50)	0·88 (0·57-1·38)	1·2
Melbye et al., 1992	Sputum (purulent)	420	NA	NA	NA	NA	0·35	0·65	1·0 (0·5-1·8)	NA	NA

Author, Year	Index test	Total population	True positive	False negative	False positive	True negative	Sensitivity (95% CI)	Specificity (95% CI)	Positive likelihood ratio (95% CI)	Negative likelihood ratio (95% CI)	OR
Steurer et al., 2011	Sputum (Muco-purulent)	621	62	65	240	254	0·49 (0·40-0·57)	0·51 (0·47-0·56)	1·00 (0·82-1·23)	0·99 (0·82-1·20)	1·00
van Vugt et al., 2013	Phlegm	2818	120	20	2119	559	1·00 (0·96-1·0)	0·21 (0·19-0·22)	1·26 (1·24-1·29)	0·00	NA
**Bloody sputum**											
Flanders et al., 2004	Bloody sputum	168	6	14	21	127	0·30 (0·14-0·52)	0·86 (0·79-0·91)	2·11 (0·97-4·60)	0·82 (0·61-1·09)	2·6
Steurer et al., 2011	Bloody sputum	621	15	112	27	467	0·12 (0·07-0·18)	0·95 (0·92-0·96)	2·16 (1·19-3·94)	0·93 (0·87-1·00)	2·3
**Dyspnea**											
Ebrahimzadeh, et al., 2015	Dyspnea	840	323	97	180	240	0·77 (0·73-0·81)	0·57 (0·52-0·62)	1·79 (1·59-2·03)	0·40 (0·33-0·49)	4·4
Flanders et al., 2004	Dyspnea	168	14	6	71	77	0·70 (0·48-0·85)	0·52 (0·44-0·60)	1·46 (1·05-2·03)	0·58 (0·29-1·15)	2·5
Hopstaken et al., 2003	Dyspnea	243	23	9	165	46	0·72 (0·55-0·84)	0·22 (0·17-0·28)	0·92 (0·73-1·16)	1·29 (0·70-2·37)	0·7
Melbye et al., 1992	Dyspnea	420	NA	NA	NA	NA	0·85	0·37	1·4 (1·0-1·8)	NA	NA
Signal et al., 1989	Dyspnea	255	16	24	92	123	0·40 (0·26-0·55)	0·57 (0·50-0·64)	0·94 (0·62-1·41)	1·05 (0·79-1·39)	0·9
Steurer et al., 2011	Dyspnea	621	58	69	165	329	0·46 (0·37-0·54)	0·67 (0·62-0·71)	1·37 (1·72-0·69)	0·82 (0·69-0·97)	1·7
van Vugt et al., 2013	Dyspnea	2819	96	44	1498	1181	0·69 (0·60-0·76)	0·44 (0·42-0·46)	1·23 (1·09-1·38)	0·71 (0·56-0·91)	1·7
**Chest pain**											
Ebrahimzadeh, et al., 2015	Chest pain	840	315	105	201	219	0·75 (0·71-0·79)	0·52 (0·47-0·57)	1·57 (1·40-1·76)	0·48 (0·40-0·58)	3·3
Flanders et al., 2004	Chest pain	168	9	11	33	115	0·45 (0·26-0·66)	0·78 (0·70-0·84)	2·02 (1·14-3·57)	0·71 (0·47-1·06)	2·9
Hopstaken et al., 2003	Chest pain	243	21	11	124	87	0·65 (0·48-0·80)	0·41 (0·35-0·48)	1·12 (0·85-1·47)	0·83 (0·50-1·38)	1·4
Melbye et al., 1992	Chest pain	420	NA	NA	NA	NA	0·40	0·79	1·9 (1·0-3·6)	NA	NA
Moberg et al., 2016	Chest pain	100	7	38	10	45	0·16 (0·08-0·29)	0·82 (0·70-0·90)	0·86 (0·35-2·07)	1·03 (0·87-1·23)	0·8
Signal et al., 1989	Chest pain	255	19	21	80	135	0·48 (0·33-0·63)	0·63 (0·56-0·69)	1·28 (0·88-1·85)	0·84 (0·61-1·14)	1·5
Steurer et al., 2011	Chest pain	621	46	81	133	361	0·36 (0·28-0·45)	0·73 (0·69-0·77)	1·35 (1·02-1·77)	0·87 (0·76-1·01)	1.5
van Vugt et al., 2013	Chest pain	2817	80	60	1224	1453	0·57 (0·49-0·65)	0·54 (0·52-0·56)	1·25 (1·08-1·45)	0·79 (0·65-0·96)	1·6
**Coryza**											
Flanders et al., 2004	Runny nose	168	9	11	106	42	0·45 (0·26-0·66)	0·28 (0·22-0·36)	0·63 (0·38-1·03)	1·94 (1·21-3·11)	0·3

Author, Year	Index test	Total population	True positive	False negative	False positive	True negative	Sensitivity (95% CI)	Specificity (95% CI)	Positive likelihood ratio (95% CI)	Negative likelihood ratio (95% CI)	OR
Melbye et al., 1992	Coryza	420	NA	NA	NA	NA	0·65	0·18	0·8 (0·6-1·1)	NA	NA
van Vugt et al., 2013	Runny nose	2819	79	61	1932	747	0·56 (0·48-0·64)	0·28 (0·27-0·30)	0·78 (0·68-0·91)	1·56 (1·28-1·91)	0·5
**Sore throat**											
Flanders et al., 2004	Sore throat	168	9	11	100	48	0·45 (0·26-0·66)	0·32 (0·25-0·40)	0·67 (0·41-1·10)	1·70 (1·07-2·69)	0·4
Melbye et al., 1992	Sore throat	420	NA	NA	NA	NA	0·45	0·25	0·6 (0·4-1·0)	NA	NA
**Fatigue**											
Flanders et al., 2004	Fatigue	168	14	6	109	39	0·70 (0·48-0·85)	0·26 (0·20-0·34)	0·95 (0·70-1·29)	1·14 (0·55-2·34)	0·8
Melbye et al., 1992	Fatigue	420	NA	NA	NA	NA	1·00	0·11	1·1 (1·0-1·3)	NA	NA
**Myalgia/arthralgia**											
Flanders et al., 2004	Myalgia	168	14	6	79	69	0·7 (0·48-0·85)	0·47 (0·39-0·55)	1·31 (0·95-1·81)	0·64 (0·32-1·29)	2·0
Melbye et al., 1992	Myalgia/arthralgia	420	NA	NA	NA	NA	0·55	0·45	1·0 (0·7-1·5)	NA	NA
**Wheezing**											
Flanders et al., 2004	Wheezing	168	11	9	48	100	0·55 (0·34-0·74)	0·68 (0·60-0·75)	1·70 (1·07-2·69)	0·67 (0·41-1·10)	2·5
Steurer et al., 2011	Wheezing	621	24	103	85	409	0·19 (0·13-0·27)	0·83 (0·79-0·86)	1·10 (0·73-1·65)	0·98 (0·89-1·08)	1·1
**Diarrhoea**											
Hopstaken et al., 2003	Diarrhoea	243	6	26	13	198	0·19 (0·09-0·35)	0·94 (0·90-0·96)	3·04 (1·25-7·43)	0·87 (0·73-1·03)	3·5
van Vugt et al., 2013	Diarrhoea	2818	15	125	184	2494	0·11 (0·07-0·17)	0·93 (0·92-0·94)	1·56 (0·95-2·57)	0·96 (0·90-1·02)	1·6

Signs

Author, Year	Index test	Total population	True positive	False negative	False positive	True negative	Sensitivity (95% CI)	Specificity (95% CI)	Positive likelihood ratio (95% CI)	Negative likelihood ratio (95% CI)	OR
**Temperature**											
Ebrahimzadeh, et al., 2015	temperature ≥38°C	840	285	135	68	352	0·68 (0·63-0·72)	0·84 (0·80-0·87)	4·19 (3·34-5·26)	0·38 (0·33-0·44)	10·9
Flanders et al., 2004	temperature ≥37.8°C	155	10	9	19	117	0·53 (0·32-0·73)	0·86 (0·79-0·91)	3·77 (2·08-6·84)	0·55 (0·34-0·89)	6·8
Moberg et al., 2016	temperature >38°C	100	15	30	6	49	0·33 (0·21-0·48)	0·89 (0·78-0·95)	3·06 (1·29-7·22)	0·75 (0·60-0·94)	4·1
Holm, Nexoe, et al., 2007	temperature ≥38°C	354	14	33	33	274	0·30 (0·19-0·44)	0·89 (0·85-0·92)	2·8 (1·61-4·78)	0·79 (0·65-0·95)	3·5
Hopstaken et al., 2003	temperature ≥38°C	243	13	19	45	166	0·41 (0·26-0·58)	0·79 (0·73-0·84)	1·91 (1·16-3·12)	0·76 (0·56-1·01)	2·5
Hopstaken et al., 2009	temperature ≥38°C	95	4	7	14	70	0·36 (0·15-0·65)	0·83 (0·74-0·90)	2·18 (0·87-5·46)	0·76 (0·8-1.21)	2.9
Melbye et al., 1992	temperature ≥37.5°C	420	NA	NA	NA	NA	0·50	0·70	1·7 (1·0-2·9)	NA	NA
Nolt et al., 2007	temperature ≥38.6°C	4464	NA	NA	NA	NA	NA	NA	4·15 (3·28-5·26)	NA	NA
van Vugt et al., 2013	temperature >37.8°C	2793	22	118	136	2517	0·15 (0·11-0·23)	0·95 (0·94-0·96)	3·01 (2·02-4·65)	0·89 (0·83-0·96)	3·5
**Pulse rate**											
Ebrahimzadeh, et al., 2015	pulse rate >100 min^{-1}	840	231	189	63	357	0·55 (0·50-0·60)	0·85 (0·81-0·88)	3·67 (2·87-4·68)	0·53 (0·47-0·59)	6·9
Flanders et al., 2004	pulse rate ≥100 min^{-1}	157	11	9	26	111	0·55 (0·34-0·74)	0·81 (0·74-0·87)	2·90 (1·71-4·91)	0·56 (0·34-0·91)	5·2
Moberg et al., 2016	pulse rate >100 min^{-1}	100	13	32	15	40	0·29 (0·18-0·43)	0·73 (0·60-0·83)	1·06 (0·56-1·99)	0·98 (0·76-1·25)	1·1
Holm, Nexoe, et al., 2007	pulse rate >100 min^{-1}	364	12	36	31	285	0·25 (0·15-0·39)	0·90 (0·86-0·93)	2·55 (1·41-4·61)	0·83 (0·70-0·98)	3·1
Nolt et al., 2007	pulse rate ≥120 min^{-1}	4464	NA	NA	NA	NA	NA	NA	4·55 (3·57-5·79)	NA	NA

Author, Year	Index test	Total population	True positive	False negative	False positive	True negative	Sensitivity (95% CI)	Specificity (95% CI)	Positive likelihood ratio (95% CI)	Negative likelihood ratio (95% CI)	OR
van Vugt et al., 2013	pulse rate >100 min^{-1}	2784	17	123	94	2550	0·12 (0·08-0·19)	0·96 (0·96-0·97)	3·42 (2·10-5·56)	0·91 (0·86-0·97)	3·7
**Respiratory rate**											
Ebrahimzadeh, et al., 2015	respiratory rate ≥20 min^{-1}	840	252	168	44	376	0·60 (0·55-0·65)	0·89 (0·86-0·92)	5·73 (4·28-7·66)	0·45 (0·40-0·51)	12·8
Flanders et al., 2004	respiratory rate >=24 min^{-1}	121	7	11	2	101	0·39 (0·20-0·61)	0·98 (0·93-0·99)	20·03 (4·52-88·83)	0·62 (0·43-0·90)	32·1
Moberg et al., 2016	respiratory rate >20 min-^{1}	100	26	19	29	26	0·58 (0·43-0·71)	0·47 (0·35-0·60)	1·10 (0·77-1·56)	0·89 (1·39-0·56)	1·2
Holm, Nexoe, et al., 2007	respiratory rate ≥22 min^{-1}	364	24	24	65	251	0·50 (0·36-0·63)	0·79 (0·75-0·83)	2·43 (1·7-3·47)	0·63 (0·47-0·84)	3·9
Hopstaken et al., 2003	respiratory rate >20 min-^{1}	243	1	31	8	203	0·03 (0·00-0·16)	0·96 (0·93-0·98)	0·82 (0·11-6·37)	1·01 (0·94-1·08)	0·8
Nolt et al., 2007	respiratory rate ≥30 min-^{1}	4464	NA	NA	NA	NA	NA	NA	9.42 (6.07-14.61)	NA	NA
van Vugt et al., 2013	respiratory rate >24 min^{-1}	2753	6	134	49	2564	0·04 (0·02-0·09)	0·98 (0·98-0·99)	2·29 (1·00-5·24)	0·98 (0·94-1·01)	2·4
**O2 saturation**											
Flanders et al., 2004	O2 saturation ≤93%	112	6	14	1	91	0·30 (0·15-0·52)	0·99 (0·94-1·00)	27·6 (3·51-216·77)	0·71 (0·53-0·94)	39·0
Moberg et al., 2016	O2 saturation <95%	100	14	31	12	43	0·31 (0·19-0·46)	0·78 (0·66-0·87)	1·43 (0·74-2·77)	0·88 (0·69-1·12)	1·6
Holm, Nexoe, et al., 2007	O2 saturation <95%	357	25	23	61	248	0·52 (0·38-0·66)	0·80 (0·75-0·84)	2·64 (1·86-3·75)	0·60 (0·44-0·81)	4·4
Nolt et al., 2007	O2 saturation <90%	4464	NA	NA	NA	NA	NA	NA	10·91 (7·48-15·91)	NA	NA
**Crackles**											
Moberg et al., 2016a	Crackles	100	21	24	27	28	0·47 (0·33-0·61)	0·51 (0·38-0·64)	0·95 (0·63-1·44)	1·05 (0·72-1·53)	0·91
Moberg et al., 2016b	Rales	100	15	8	30	47	0·33 (0·21-0·48)	0·85 (0·74-0·92)	2·29 (1·07-4·91)	0·78 (0·62-0·99)	2·9
Hopstaken et al., 2003	Crackles	243	9	23	41	170	0·28 (0·16-0·45)	0·81 (0·75-0·85)	1·45 (0·78-2·69)	0·89 (0·71-1·12)	1·6

Author, Year	Index test	Total population	True positive	False negative	False positive	True negative	Sensitivity (95% CI)	Specificity (95% CI)	Positive likelihood ratio (95% CI)	Negative likelihood ratio (95% CI)	OR
Melbye et al., 1992	Crackles	420	NA	NA	NA	NA	0·20	0·94	4·4 (1·4-13·6)	NA	NA
Signal et al., 1989	Crackles	255	16	24	49	166	0·40 (0·26-0·55)	0·77 (0·71-0·82)	1.76 (1·12-2.76)	0·78 (0·59-1.0)	2·26
van Vugt et al., 2013	Crackles	2807	45	95	220	2447	0·32 (0·25-0·40)	0·92 (0·91-0·93)	3·90 (2·97-5·11)	0·74 (0·66-0·83)	5·3
Flanders et al., 2004	Rales	166	10	10	5	141	0·50 (0·30-0·70)	0·97 (0·92-0·99)	14·60 (5·55-38·38)	0·52 (0·80-8·08)	28.2
**Decreased breath sounds**											
Flanders et al., 2004	Decreased breath sounds	166	12	8	15	131	0·60 (0·39-0·78)	0·90 (0·84-0·94)	5·84 (3·21-10·62)	0·45 (0·26-0·77)	13·1
Moberg et al., 2016	Decreased breath sounds	100	9	36	14	41	0·20 (0·11-0·34)	0·75 (0·62-0·84)	0·79 (0·38-1·65)	1·07 (0·87-1·33)	0·73
Melbye et al., 1992	Decreased breath sounds	420	NA	NA	NA	NA	0·15	0·95	3·2 (1·0-10·0)	NA	NA
Steurer et al., 2011	Decreased breath sounds	621	31	96	43	451	0·24 (0·178-0·33)	0·91 (0·88-0·93)	2·8 (1·85-4·26)	0·83 (0·75-0·92)	3·39
van Vugt et al., 2013	Decreased breath sounds	2805	31	109	331	2334	0·22 (0·16-0·30)	0·88 (0·86-0·89)	1·78 (1·30-2·47)	0·89 (0·81-0·97)	2·0
**Bronchial breathing**											
Hopstaken et al., 2003	Bronchial breathing	243	11	21	53	158	0·34 (0·20-0·52)	0·75 (0·69-0·80)	1·37 (0·80-2·33)	0·88 (0·67-1·14)	1·6
Steurer et al., 2011	Bronchial breathing	621	24	103	28	466	0·19 (0·13-0·27)	0·94 (0·92-0·96)	3·33 (2·00-5·55)	0·86 (0·79-0·94)	3·9
**Wheezes**											
Flanders et al., 2004	Wheezing	166	5	15	28	118	0·25 (0·11-0·47)	0·80 (0·74-0·86)	1·30 (0·57-2·99)	0·93 (0·71-1·21)	1·4
Melbye et al., 1992	Wheezing	420	NA	NA	NA	NA	0·15	0·85	1·0 (0·3-2·8)	NA	NA
Signal et al., 1989	Wheezing	255	11	29	47	168	0·28 (0·16-0·43)	0·78 (0·72-0·83)	1·26 (0·72-2·21)	0·93 (0·76-1·14)	1·36
**Dullness on percussion**											
Moberg et al., 2016	Dullness on percussion	100	5	40	10	45	0·11 (0·05-0·24)	0·82 (0·70-0·90)	0·61 (0·23-1·66)	1·09 (0·92-1·28)	0·6

Author, Year	Index test	Total population	True positive	False negative	False positive	True negative	Sensitivity (95% CI)	Specificity (95% CI)	Positive likelihood ratio (95% CI)	Negative likelihood ratio (95% CI)	OR
Hopstaken et al., 2003	Dullness on percussion	243	0	32	11	200	0·00	0·95 (0·90-0·97)	0·00	1·10	0.0
Melbye et al., 1992	Dullness on percussion	420	NA	NA	NA	NA	0·14	0·96	4·0 (1·0-16·7)	NA	NA
Steurer et al., 2011	Dullness on percussion	621	25	102	13	481	0·20 (0·14-0·27)	0·97 (0·96-0·98)	7·48 (3·94-14·20)	0·83 (0·76-0·90)	9·1

Laboratory tests

Author, Year	Index test	Total population	True positive	False negative	False positive	True negative	Sensitivity (95% CI)	Specificity (95% CI)	Positive likelihood ratio (95% CI)	Negative likelihood ratio (95% CI)	OR
**WBC**											
Ebrahimzadeh, et al., 2015	WBC ≥10 million/ml	840	204	216	94	326	0·49 (0·44-0·53)	0·78 (0·74-0·81)	2·17 (1·78-2·66)	0·66 (0·60-0·74)	3·3
Moberg et al., 2016	WBC ≥15 million/ml	100	7	38	5	50	0·16 (0·08-0·29)	0·91 (0·80-0·96)	1·71 (0·58-5·03)	0·93 (0·80-1·08)	1·8
Holm, Nexoe, et al., 2007	WBC ≥10 million/ml	362	22	26	64	250	0·46 (0·33-0·60)	0·80 (0·75-0·84)	2·25 (1·54-3·28)	0·68 (0·52-0·9)	3·3
Melbye et al., 1992	WBC ≥10 million/ml	420	NA	NA	NA	NA	0·53	0·85	3·1 (1·7-5·7)	NA	NA
**ESR**											
Melbye et al., 1992	ESR≥35mm/h	420	NA	NA	NA	NA	0·50	0·89	4·3 (2·4-7·8)	NA	NA
**Procalcitonin**											
Holm, Pedersen, et al., 2007	PCT >0.25ng/ml	357	11	36	4	306	0·23 (0·14-0·37)	0·99 (0·97-0·99)	18·14 (6·02-54·63)	0·78 (0·66-0·91)	23·4
van Vugt et al., 2013	PCT >0.25ng/ml	2664	20	120	140	2414	0·14 (0·09-0·21)	0·95 (0·94-0·95)	2·61 (1·68-4·03)	0·91 (0·85-0·97)	2.9
Holm, Pedersen, et al., 2007	PCT >0.50ng/ml	357	8	39	0	310	0·17 (0·09-0·30)	1·00 (0·99-1.00)	NA	0·83 (0·73-0·94)	NA
van Vugt et al., 2013	PCT >0.50ng/ml	2664	14	126	66	2458	0·10 (0·06-0·16)	0·97 (0·97-0·98)	3·8 (2·20-6·64)	0·92 (0·87-0·98)	4·1
**CRP**											
Holm, Nexoe, et al., 2007	CRP≥20mg/l	363	35	13	110	205	0·73 (0·59-0·83)	0·65 (0·60-0·70)	2·09 (1·66-2·63)	0·42 (0·26-0·68)	5·0
Hopstaken et al., 2009	CRP>20mg/l	95	11	0	41	43	1·00 (0·74-1·0)	0·51 (0·41-0·62)	2·05 (1·65-2·55)	0·00	NA
van Vugt et al., 2013	CRP>20mg/l	2678	85	55	641	1897	0·61 (0·52-0·68)	0·75 (0·73-0·76)	2·40 (2·07-2·79)	0·53 (0·43-0·65)	4·6
Moberg et al., 2016	CRP>50mg/l	100	37	8	20	35	0·82 (0·69-0·91)	0·64 (0·50-0·75)	2·26 (1·55-3·29)	0·28 (0·14-0·54)	8·1

Author, Year	Index test	Total population	True positive	False negative	False positive	True negative	Sensitivity (95% CI)	Specificity (95% CI)	Positive likelihood ratio (95% CI)	Negative likelihood ratio (95% CI)	OR
Melbye et al., 1992	CRP≥50mg/l	420	NA	NA	NA	NA	0·50	0·90	4.8 (2·70-8·60)	NA	NA
van Vugt et al., 2013	CRP>50mg/l	2678	58	82	197	2341	0·41 (0·34-0·50)	0·92 (0·91-0·93)	5·34 (4·21-6·77)	0·63 (0·55-0·73)	8·4
Flanders et al., 2004	CRP≥100mg/l	168	7	13	1	147	0·35 (0·18-0·57)	0·99 (0·96-1·0)	51·80 (6·72-399·41)	0·65 (0·47-0·90)	79·15
Hopstaken et al., 2009	CRP>100mg/l	95	9	2	13	71	0·82 (0·52-0·95)	0·85 (0·75-0·91)	5·29 (2·98-9·37)	0·21 (0·06-0·76)	24·58
Steurer et al., 2011	CRP≥100mg/l	621	73	54	61	433	0·57 (0·49-0·66)	0·88 (0·84-0·90)	4·66 (3·52-6·15)	0·49 (0·40-0·60)	9·6
van Vugt et al., 2013	CRP>100mg/l	2678	34	106	62	2476	0·24 (0·18-0·32)	0·98 (0·97-0·98)	9·94 (6·78-14·57)	0·78 (0·71-0·85)	12·8
**Lipopolysaccharide binding protein (LBP)**											
Hopstaken et al., 2009	LBP>10	95	11	0	56	28	1·00 (0·74-1·0)	0·33 (0·24-0·44)	1·5 (1·29-1·75)	0·00	NA
	LBP>20	95	9	2	17	67	0·81 (0·52-0·95)	0·79 (0·70-0·87)	4·04 (2·43-6·71)	0·23 (0·07-0·80)	17·7
	LBP>30	95	8	3	8	76	0·73 (0·43-0·90)	0·90 (0·82-0·95)	7·64 (3·6-16·20)	0·30 (0·12-0·79)	25·33
**Fibrinogen**											
Hopstaken et al., 2009	Fibrinogen>4	95	10	1	38	46	0·90 (0.62-0.98)	0·55 (0·44-0·65)	2·01 (1·49-2·71)	0·17 (0·03-1·09)	12·11
	Fibrinogen>5	95	9	2	9	75	0·81 (0·52-0·95)	0·89 (0·81-0·94)	7·63 (3·88-15·03)	0·20 (0·06-0·72)	37·5
